# Application of Porous Copper-Based Interconnect Materials in Electronic Packaging: A Brief Review

**DOI:** 10.3390/ma19183937

**Published:** 2026-09-16

**Authors:** Jiahao Liu, Lijin Qiu, Peizhong Wang, Feiyang Wang, Jixi Huang, Hongtao Chen, Hao Zhao, Fangzhou Chen

**Affiliations:** 1 Department of Integrated Circuit, Harbin Institute of Technology (Shenzhen), Shenzhen 518055, China; ljh071408@163.com (J.L.); 24s055026@stu.hit.edu (L.Q.); 19567086072@163.com (P.W.); 13366753516@163.com (F.W.); 2023313528@stu.hit.edu.cn (J.H.); 2 Science and Technology on Reliability Physics and Application of Electronic Component Laboratory, China Electronic Product Reliability and Environmental Testing Research Institute, Guangzhou 511370, China

**Keywords:** porous Cu, power device packaging, fabrication methods, interconnection techniques, long-term reliability

## Abstract

Driven by the rapid development of deep-space exploration, artificial intelligence, and new energy vehicles, the integration density of power devices has been significantly improved, creating an urgent demand for electronic packaging interconnection materials with low thermal resistance, high reliability, and thermomechanical stress resistance. Porous metals have become a major research focus for packaging interconnection materials owing to their high specific surface area; controllable porous structure; and excellent thermal, electrical, and mechanical properties. Among various porous metals, porous metal has attracted extensive attention in the field of electronic packaging interconnection due to its low cost, high thermal conductivity, superior wettability, and good processability. This paper systematically reviews the research progress of porous copper (Cu)-based interconnection materials in electronic packaging, covering the preparation methods, interconnection methods and reliability of porous Cu-based interconnection materials. In addition, the existing challenges in the preparation, interconnection processes, and reliability evaluation are summarized, which provides clear research directions for the structural optimization and engineering application of such materials in the future.

## 1. Introduction

With the rapid development of deep-space exploration, artificial intelligence, and new energy vehicles, the power density of electronic devices has increased significantly, resulting in a sharp rise in chip junction temperatures during service. However, conventional interconnection materials are limited by their intrinsic properties and are prone to failure under high-temperature conditions, such as melting, delamination, increased thermal resistance, and degradation of mechanical strength [1,2,3]. To meet the stringent requirements for high-reliability interconnection, novel materials with superior comprehensive performance are urgently needed, and porous metals perfectly fit this application scenario. Porous metals consist of an interconnected metallic ligament and a continuous pore network, forming a three-dimensional bicontinuous matrix, which exhibits low density, high specific surface area, excellent thermal conductivity, and favorable mechanical properties [4,5,6,7]. Ashby et al. demonstrated that porous metals, owing to their efficient heat transfer pathways and designable pore structures, can effectively alleviate thermal mismatch and enhance interfacial stability in electronic packaging [8]. Moreover, Zhang et al. emphasized that interconnection materials should possess sufficient compliance and deformability to release thermomechanical stresses generated during service [9]. Currently, porous metals such as porous gold (Au), silver (Ag), and nickel (Ni) have been investigated for interconnection bondlines. Oppermann et al. employed porous Au bumps to achieve low-temperature flip-chip bonding; however, this method suffers from complex processing and high costs [10]. Chen et al. proposed a rapid large-area bonding approach using an Ag porous sheet, achieving a shear strength of 82.1 MPa. However, the reliance on Ag still raises concerns about cost and stability for long-term high-temperature applications [11]. Xiao et al. introduced a Ni-foam/Sn composite interlayer with ultrasonic assistance to achieve strong Cu alloy joints; however, the excessive growth of loose porous IMC on Ni ligaments and the dissolution of Ni at longer soldering times remain concerns for joint reliability [12]. In comparison, porous copper (Cu) has attracted considerable attention due to its low cost, high thermal conductivity, excellent wettability, corrosion resistance, and good processability. More importantly, porous Cu-based interconnect bondlines exhibit superior mechanical, thermal, and electrical properties, making them promising candidates for advanced electronic packaging applications [13,14,15].

## 2. Fabrication Technologies of Porous Cu

The preparation methods for porous Cu are mainly categorized into four types, namely electrodeposition, dealloying, powder sintering, and the redox method.

**Figure 1:**
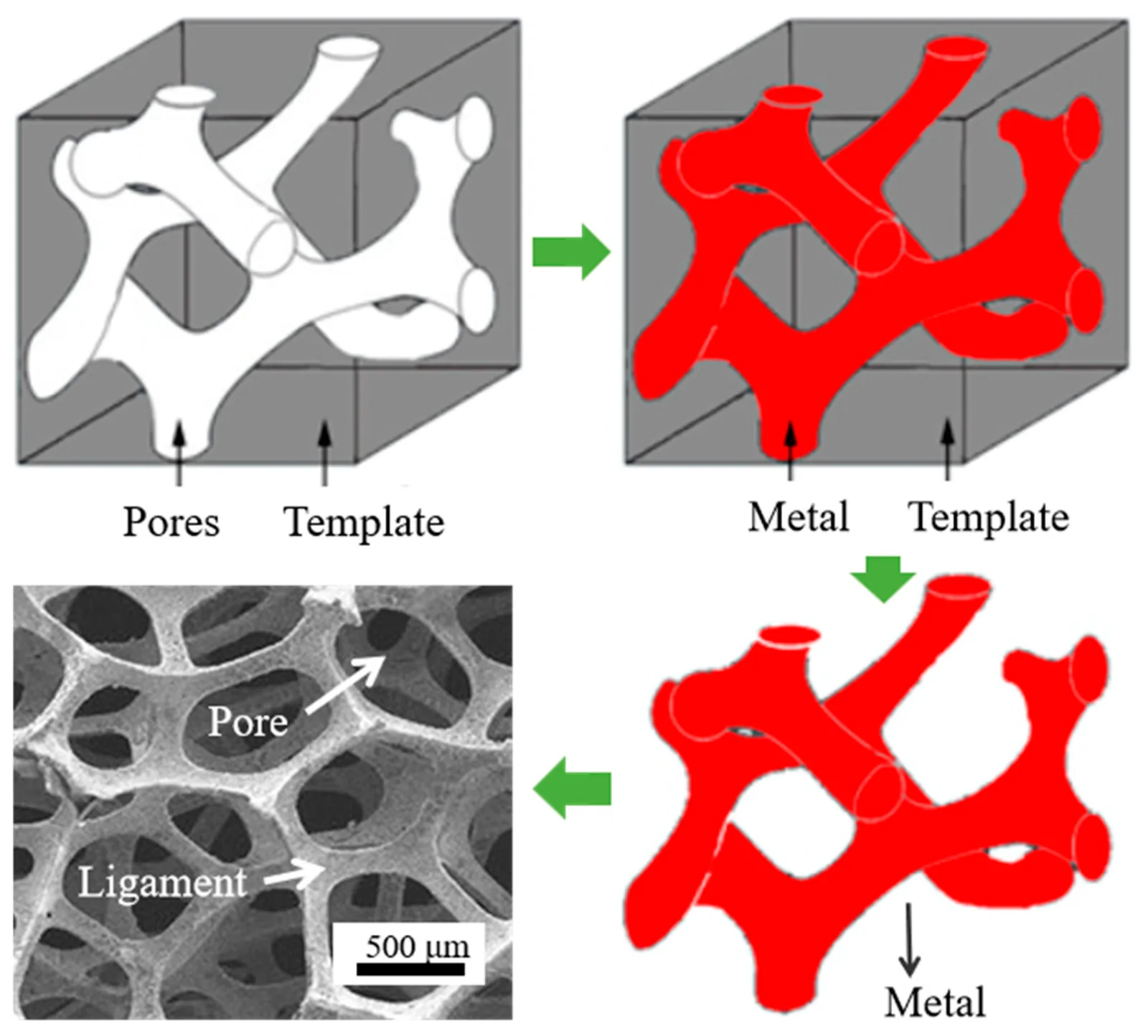
Schematic diagrams and microstructures of porous Cu based on electrochemical deposition method.

As shown in Figure 1, the electrodeposition method mainly utilizes the principle of electrochemical reduction. By combining polymer foams or dynamic hydrogen bubble templates to regulate the pore structure, this method can prepare porous Cu with millimeter-level pores and ligaments. Specifically, a sacrificial template with an open-cell architecture serves as a negative mold; Cu is then electrodeposited along the template’s surface, forming a Cu-template composite. Subsequent template removal via calcination or etching yields monolithic porous Cu, where the original template struts become Cu ligaments, and template pores become interconnected voids. However, this approach faces the key challenge of complex template removal processes, which risk structural damage, oxidation, or residual impurities. When using solid materials as templates, Hanim et al. and Liu et al. used polyurethane foam templates to prepare large pore skeletons with pore sizes of 140–680 µm and 50–200 µm and corresponding ligament thicknesses of 30–170 µm [16,17]. When using dynamic bubble templates, the pores and skeletons of porous Cu can be further refined. Guo et al. used hydrogen bubbles that precipitated during the electrolysis process as a dynamic template and prepared three-dimensional porous Cu with a pore size of about 17.68 µm and a ligament thickness of about 17.70 µm [18].

**Figure 2:**
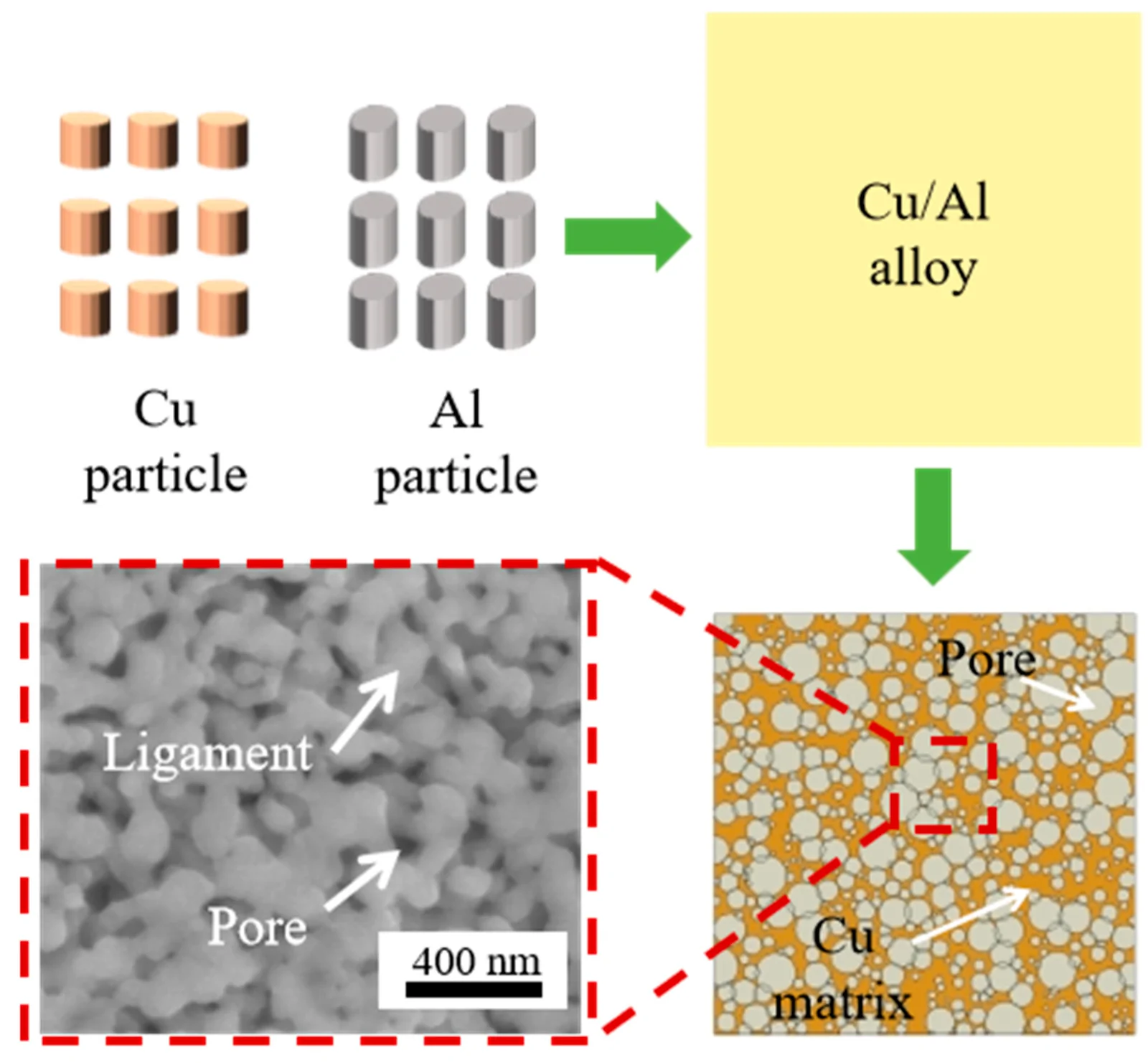
Schematic diagrams and microstructures of porous Cu based on powder sintering method.

As shown in Figure 2, the dealloying method selectively removes active components from alloy precursors (e.g., Cu-Zn, Cu-Mg, Cu-Al and Cu-Mn-Si) via chemical or electrochemical corrosion. Under specific electrolyte or electrochemical polarization conditions, active metals with lower potentials are preferentially dissolved, while chemically stable Cu atoms remain and are reconstructed via surface diffusion and atomic rearrangement, forming a continuous, interconnected three-dimensional nanoporous Cu matrix. Moreover, the pore and ligament microstructure can be accurately tuned by adjusting the alloy composition, dealloying duration, electrolyte concentration, temperature and applied voltage. A key merit of this method is the fabrication of ultra-fine nanopores with sizes ranging from tens to hundreds of nanometers, which endow porous Cu with a large specific surface area and abundant surface-active sites. Nevertheless, this method still has obvious limitations. Rapid dissolution of active components and atomic reconstruction introduce residual internal stress, resulting in high brittleness and poor mechanical robustness. Ibrahim et al. reported dealloying results for Cu-Zn alloy, obtaining extremely small pore sizes of 15–50 nm and ligaments of 15–78 nm, but there were issues with mechanical properties [19,20,21]. Subsequent research has optimized the alloy system to improve the mechanical properties of porous Cu [22,23,24,25]. Overall, uncontrolled corrosion and random atomic diffusion may cause uneven pore distribution, local ligament coarsening and residual impurities, further limiting structural stability and practical engineering applications.

**Figure 3:**
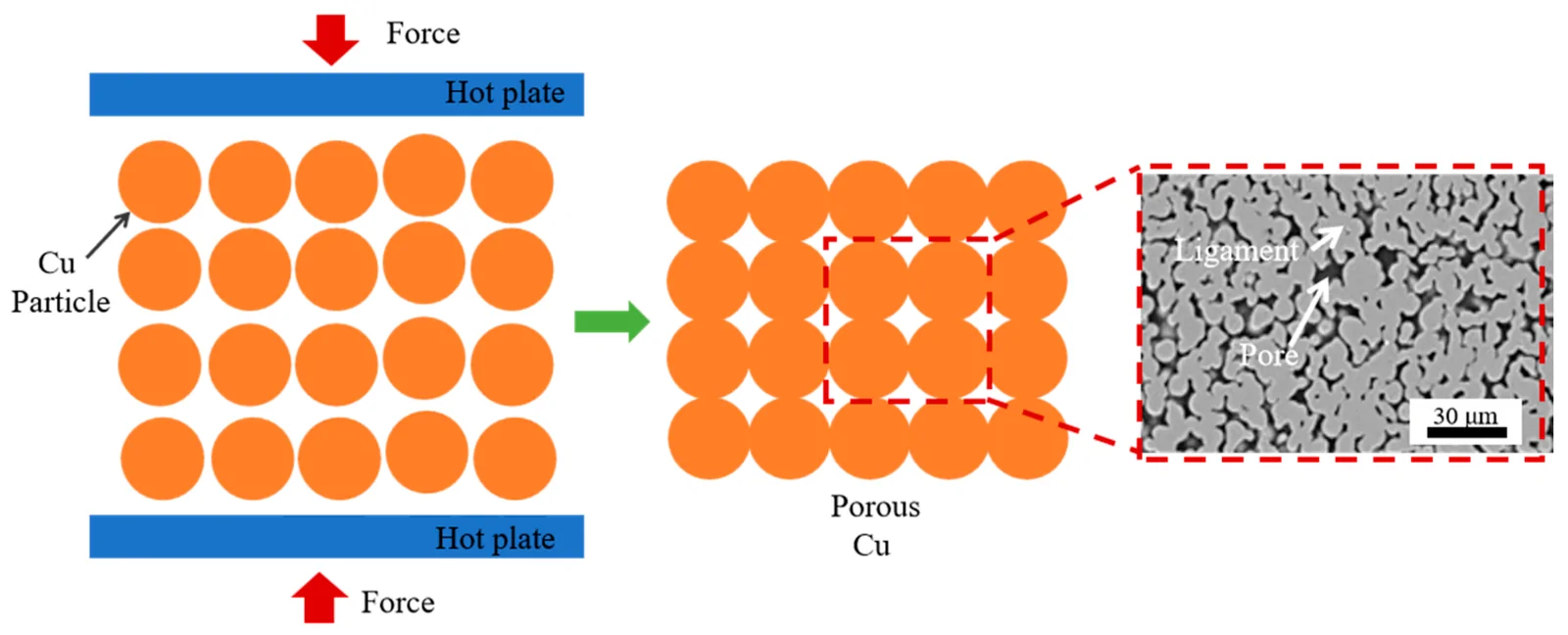
Schematic diagrams and microstructures of porous Cu based on dealloying method.

As shown in Figure 3, powder sintering is a classic and efficient method to fabricate high-purity porous Cu that uses pure Cu powder as raw material and forms stable porous structures through high-temperature atomic diffusion and sintered neck growth. Specifically, Cu powder particles only maintain loose physical contact with unstable gaps at room temperature; when sintered below the melting point of Cu, thermally activated Cu atoms diffuse intensely at particle interfaces to form sintered necks that bond discrete particles. With an elevated temperature and extended holding time, the sintered necks gradually grow and consolidate loose powder stacks into a rigid integral Cu matrix while retaining and optimizing inherent particle stacking gaps to form abundant interconnected micrometer-scale pore channels. Shao et al. obtained a porous Cu structure with a pore size of about 9.51 µm and a ligament thickness of about 18.88 µm by sintering Cu particles [26]. Similarly, Miyajima et al. prepared porous Cu with a pore size of about 5.24 µm and a ligament thickness of about 12.10 µm [27]. However, it is difficult to avoid increasing the number of Cu particles during the powder sintering process using this method, and it easily generates large pores locally, which is harmful to subsequent interconnection and service processes.

**Figure 4:**
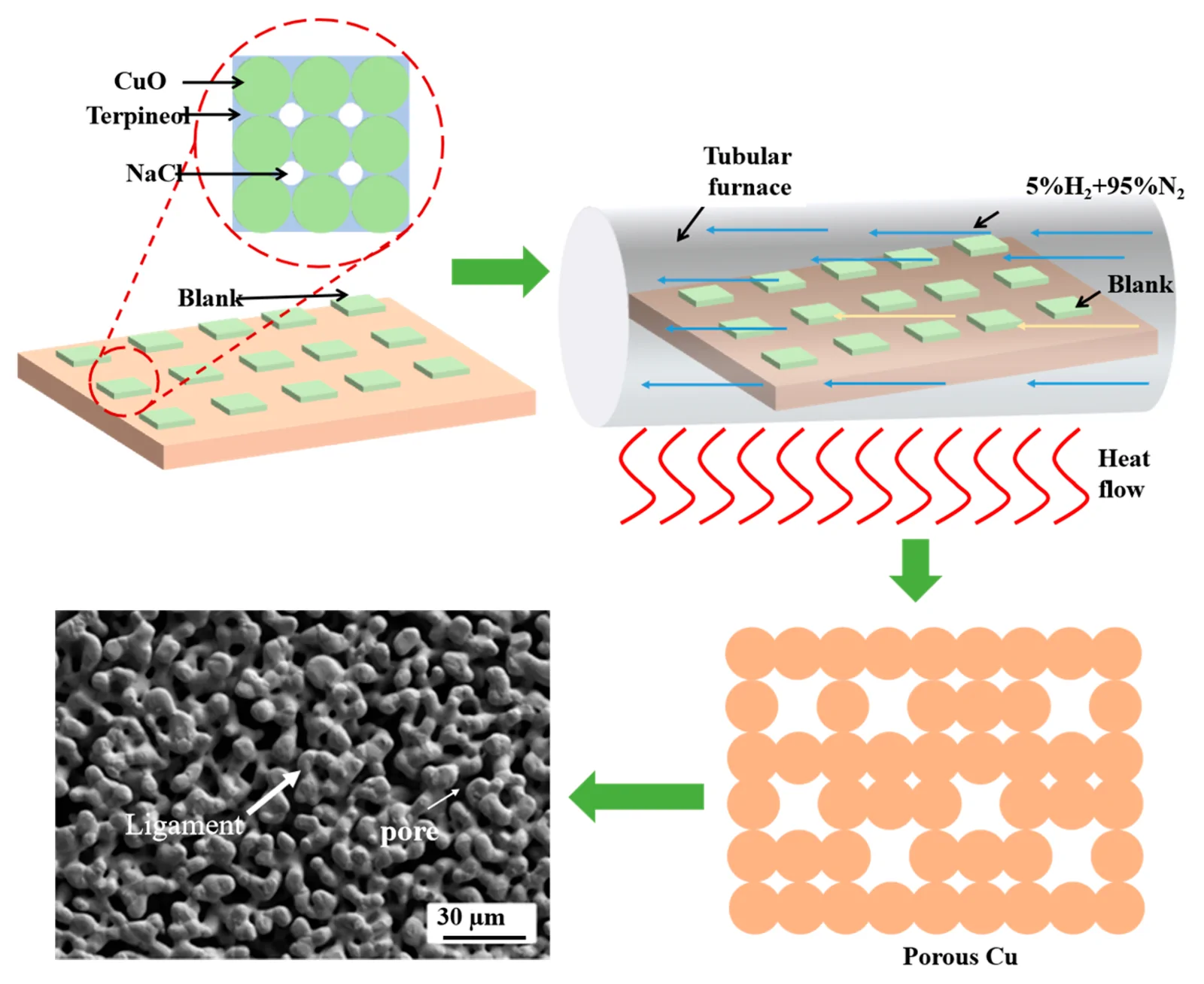
Schematic diagrams and microstructures of porous Cu based on redox-based method.

As illustrated in Figure 4, the redox-based method represents another effective route for constructing porous Cu, which relies on chemical redox reactions to modulate the morphological and structural evolution of metal precursors (e.g., copper oxides (CuO) and sulfides). Pore formation is mainly derived from structural reconstruction induced by chemical phase transformation, along with the volume shrinkage of precursor substrates or the removal of sacrificial porogens, which finally generates a stable porous Cu matrix. Lin et al. developed a sophisticated three-step sulfurization–oxidation–reduction route to fabricate a distinctive ant-hole-like porous Cu matrix. The as-prepared porous Cu possessed uniform structural parameters, with a consistent pore size and ligament thickness of approximately 3 μm, and exhibited remarkable electrochemical performance for functional applications [28]. Furthermore, our research group proposed an innovative and facile redox-assisted pore-forming strategy for customizable porous Cu fabrication. This method adopted CuO as the precursor matrix and inexpensive NaCl as a removable pore-forming agent. Benefiting from the synergistic effect of matrix redox transformation and sacrificial template pore formation, the pore size and overall porosity of the final porous Cu could be flexibly regulated by adjusting the additive amount of NaCl and sintering parameters. This novel method features low costs, simple operation and high structural controllability, and it effectively addresses the inherent limitation of uncontrollable and uneven pore structures in traditional porous Cu preparation technologies and provides a feasible and innovative technical pathway for the customized and high-performance fabrication of porous Cu [29].

As mentioned above, four representative fabrication methods for porous Cu, namely electrodeposition, dealloying, powder sintering, and redox synthesis, are evaluated in Table 1, including pore-structure control, process economics, and resulting microstructural characteristics quantified by the ImageJ2 1.5.0 software. Electrodeposition yielded macroporous structures with pore diameters spanning 17.7–680 μm and ligament thicknesses of 17.7–187.9 μm, offering low costs and favorable mechanical properties but suffering from poor shape controllability. Dealloying produced nanoporous Cu with ultra-fine pores (0.015–0.2 μm) and corresponding ligament dimensions, enabling adjustable porosity and nanoscale features but with high costs, lengthy processing times, and inherent brittleness. Powder sintering of Cu particles generated relatively large pores (5.2–9.5 μm) and thick ligaments (12.1–18.9 μm) at low cost, though it required high-temperature processing and lacked precise shape control. Redox synthesis achieved tunable porosity and pore sizes (3–100 μm) with ligament thicknesses of 3–40 μm at low material cost but was limited by prolonged processing durations and high energy consumption. These distinct capabilities and trade-offs across the four methods led to significant variations in the microstructures and performance-relevant pore/ligament dimensions of the resulting porous Cu materials.

## 3. Interconnection Technologies for Porous Cu Bondlines

Porous Cu is mainly utilized for electronic packaging through three interconnection techniques: porous Cu-reinforced solder bonding, transient liquid-phase bonding, and Cu–Cu direct bonding.

**Figure 5:**
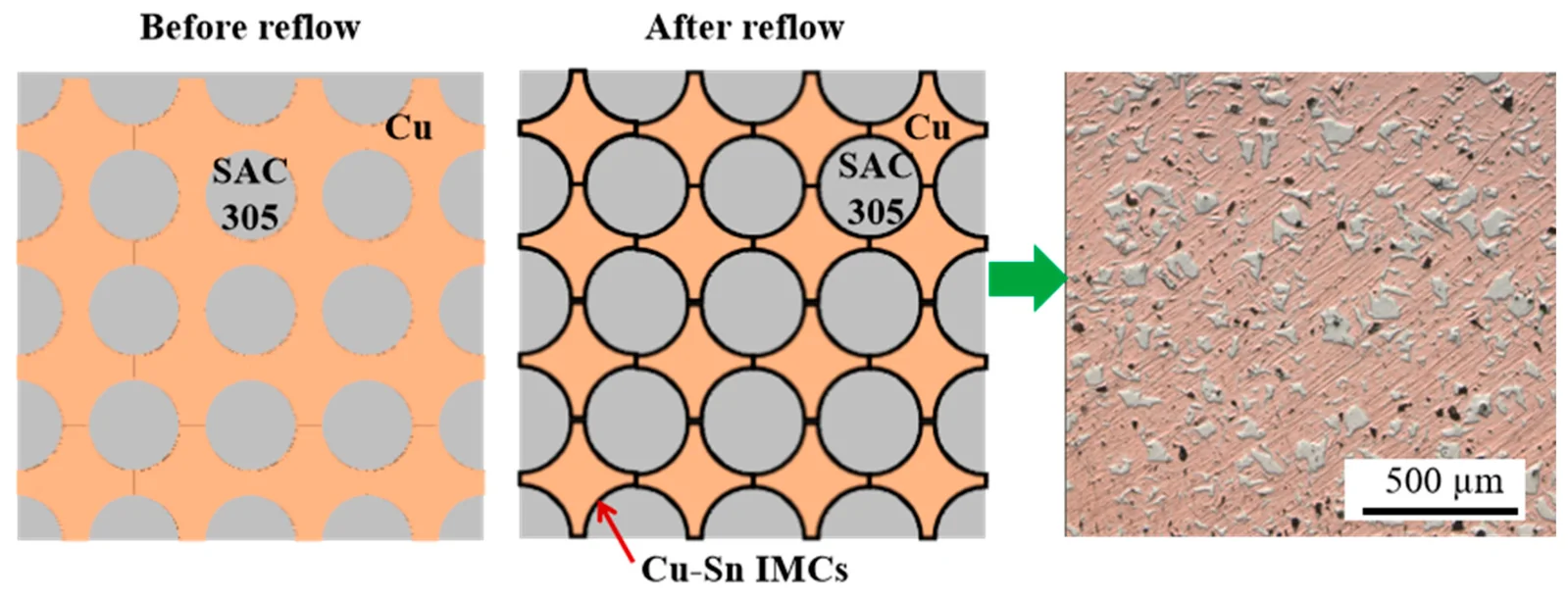
Schematic diagrams and microstructures of porous Cu interconnect bondline-based porous Cu-reinforced solder bonding method.

As shown in Figure 5, porous Cu-reinforced solder bonding is a typical composite joining method for electronic packaging. This method mainly employs large-pore-size porous Cu as the rigid matrix. In the bonding procedure, heated solder assumes a liquid state and thoroughly infiltrates into the continuous pore structure of the porous Cu matrix. The mutual embedding between the solder and porous Cu forms reliable mechanical interlocking at the joint interface. This unique structural feature effectively strengthens the interfacial shear strength of the formed bondlines. Research by Xiong et al. indicated that after bonding, the porous Cu skeleton and internal solder (SnBi,96.5Sn3.0Ag0.5Cu(SAC305)) were not completely consumed and could significantly suppress the growth of Cu-Sn intermetallic compounds (IMCs) and the segregation of Bi. Meanwhile, as a reinforcing phase, the porous Cu skeleton effectively increased the bondline shear strength to 66.9–85.2 MPa [30,31,32].

**Figure 6:**
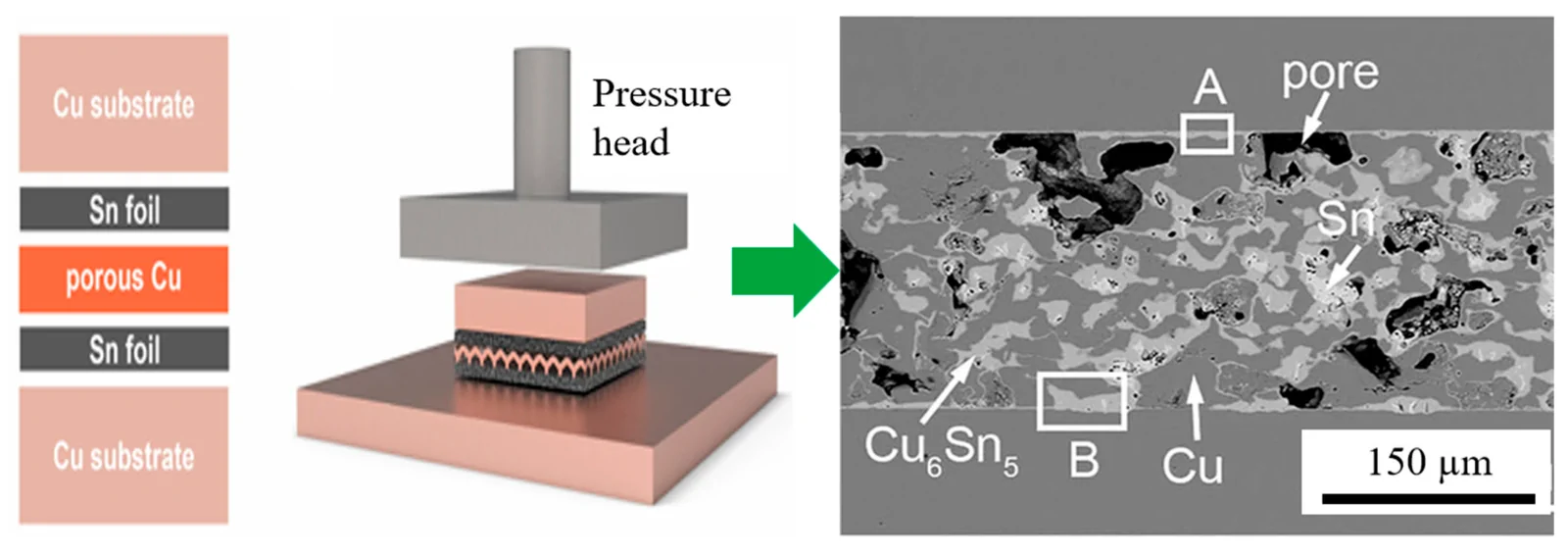
Schematic diagrams and microstructures of porous Cu interconnect bondline-based TLP method [34].

As shown in Figure 6, the transient liquid-phase bonding method uses solders such as Indium (In), Sn, and SAC305 as the low-melting-point phase and small-pore-size porous Cu as the high-melting-point phase. Driven by capillary force, the solder rapidly wets into the porous Cu matrix and reacts metallurgically to form high-melting-point Cu-Sn/Cu-In IMCs, with the low-melting-point phase being fully consumed, ultimately enabling low-temperature bonding and high-temperature operation. Castillo et al. reported that Sn-coated nanoporous Cu formed Cu–Sn IMC joints at 200–300 °C under 20 MPa. The sintered joints were dense, mainly composed of Cu_3_Sn, and showed a more uniform structure than pure porous Cu joints [33]. Similarly, Wang et al. reported that the low-melting-point Sn phase inside porous Cu was completely consumed after only 10 min of reflow at 250 °C, with its melting point increased to 415 °C. Furthermore, high-temperature shear testing of the interconnection bondlines at 300 °C yielded a shear strength as high as 73 MPa [34].

**Figure 7:**
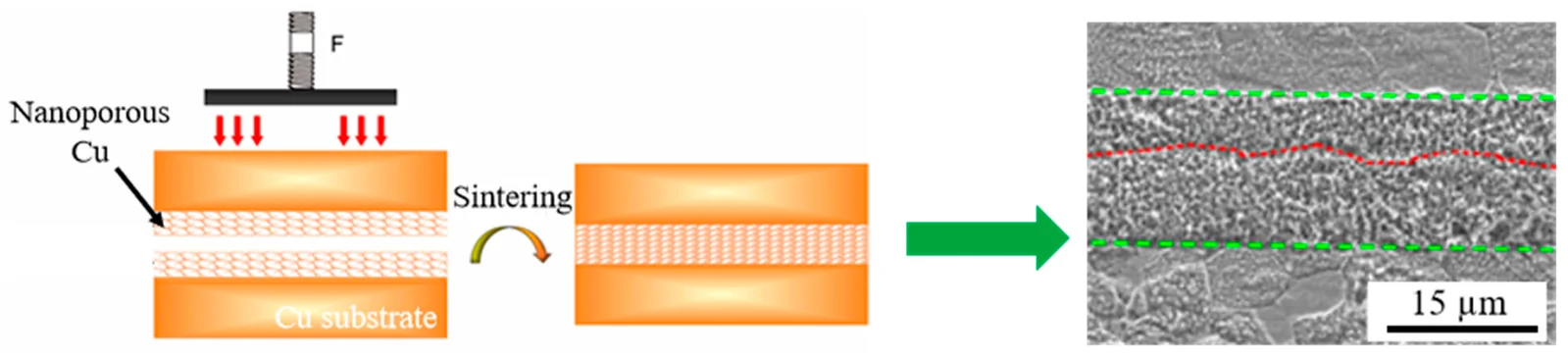
Schematic diagrams and microstructures of porous Cu interconnect bondline-based TLP method [35].

As shown in Figure 7, the Cu–Cu bonding technique refers to a solder-free pure Cu interconnection process widely applied in high-density electronic packaging. Porous Cu with nano- or submicron-scale pores is positioned between two bonding pads, followed by hot-press sintering under high temperature and a protective gas environment. Driven by the size effect of microscale pores, efficient Cu atomic thermal diffusion occurs at the contact interface between the porous Cu and substrates. The bondline fuses integrally at the interface and constructs a monolithic Cu-Cu interconnection, which overcomes the performance limitations of conventional solder joints. Research by Hang et al. demonstrated that Cu–Cu interconnection can be achieved after bonding at 150 °C and 3 MPa for 10 min. When the bonding conditions were increased to 250 °C, 3 MPa for 30 min, the Cu–Cu bondlines became almost indistinguishable, and mechanical performance was further improved, though slightly lower than that of porous Cu composite bondlines formed by transient liquid-phase bonding [35]. In addition, results from Wang et al. showed that Cu–Cu interconnection bondlines feature high thermal conductivity, high electrical conductivity, low thermal resistance, and excellent mechanical properties, meeting the requirements for high-temperature service [36,37,38,39].

As mentioned above, three representative interconnection methods for porous Cu bondlines are compared in Table 2. Porous Cu-reinforced solder bonding, using SnBi or SAC305 solders, achieved shear strengths of 66.9–85.3 MPa with inhibited IMC growth but suffered from poor high-temperature resistance and voids. TLP bonding yielded high remelting-temperature bondlines (up to 676 °C) and excellent impact resistance yet required complex processes, with a risk of interfacial voids. Cu–Cu bonding provided pure Cu bondlines with high thermal/mechanical properties (shear strength: ~22.0–40.0 MPa) but was limited by poor corrosion resistance and high processing difficulty. These trade-offs led to significant differences in the reliability and applicability of the resulting bondlines.

## 4. Reliability of Porous Cu Composite Bondlines

**Figure 8:**
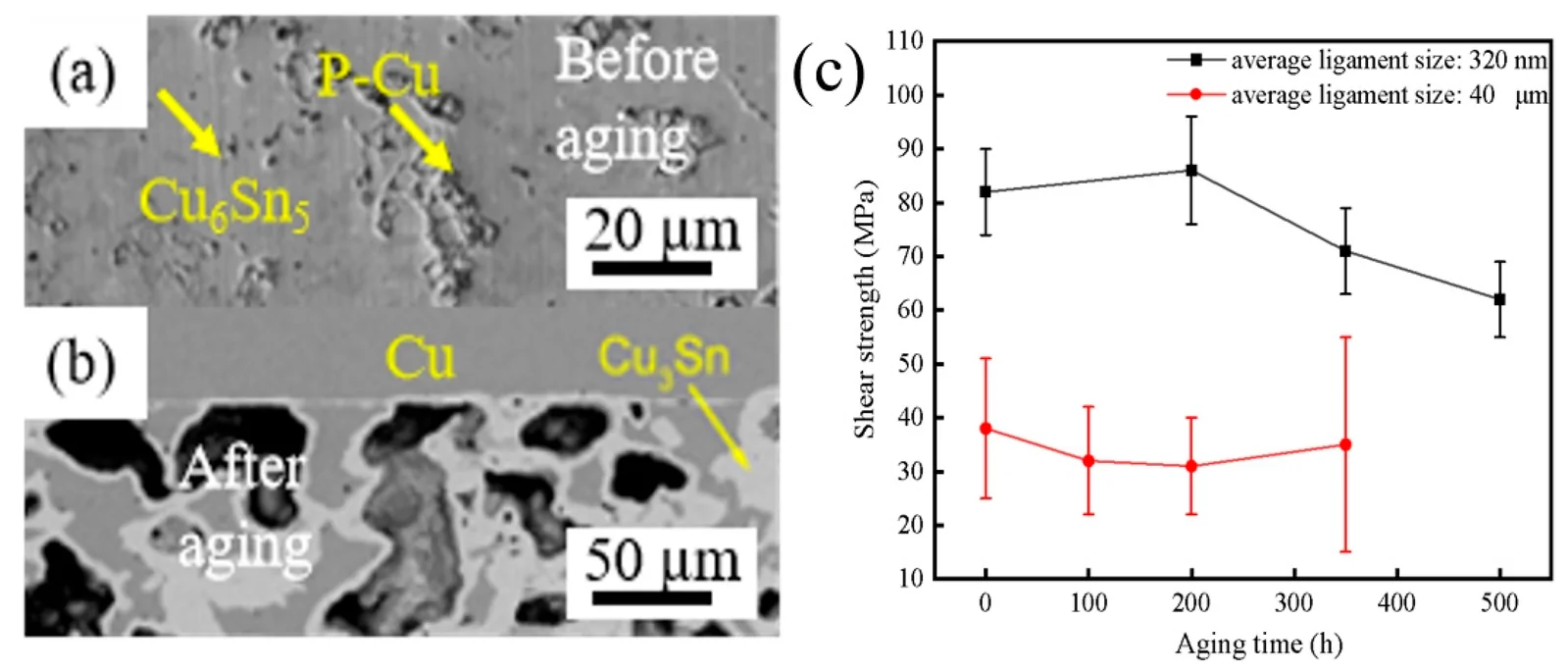
Microstructure and performance evolution of porous Cu-based interconnect bondlines before and after aging: (**a**) Microstructure before aging; (**b**) microstructure after aging; (**c**) shear strength of porous Cu/SAC305 after different aging times.

Currently, research on the reliability of porous Cu interconnect bondlines mainly focuses on tests such as high-temperature aging, thermal shock, and power cycling. Among these tests, the high-temperature aging test mainly simulates the high-temperature environment of electronic devices under long-term steady-state operation and focuses on investigating the interfacial microstructure evolution and performance degradation behavior of porous Cu bondlines. Figure 8a–c show the microstructure morphology and shear strength of porous Cu before and after aging, respectively. It is worth noting that the results obtained by different scholars indicate that discrepancies exist in the evolution laws of the microstructure morphology and interconnection strength of bondlines. Su et al. conducted aging tests on porous Cu (average ligament size: 320 nm)/SAC305 interconnect bondlines. The results showed that no obvious change in the microstructure morphology or significant variation in the shear strength of the bondlines was observed during the aging process, as the porous Cu matrix in the bondlines was not completely consumed [39,40]. In contrast, Wang, Liu et al. performed aging tests on porous Cu (average ligament size: 40 μm)/SAC305 interconnect bondlines. With the extension of aging time, the Cu matrix within the bondlines continuously reacted with Cu_6_Sn_5_ to form Cu_3_Sn, accompanied by the formation of voids. Owing to the superior mechanical properties of Cu_3_Sn compared with Cu_6_Sn_5_, the interconnection strength of the bondlines was enhanced at the initial stage of aging, whereas with the further extension of aging time, the negative impact of voids on the interconnection strength increased continuously, leading to a sustained decline in shear strength at the later stage of aging. Overall, the porous Cu/SAC305 bondlines still maintained a high level of interconnection strength after long-term aging, which effectively prevented device failure [41].

**Figure 9:**
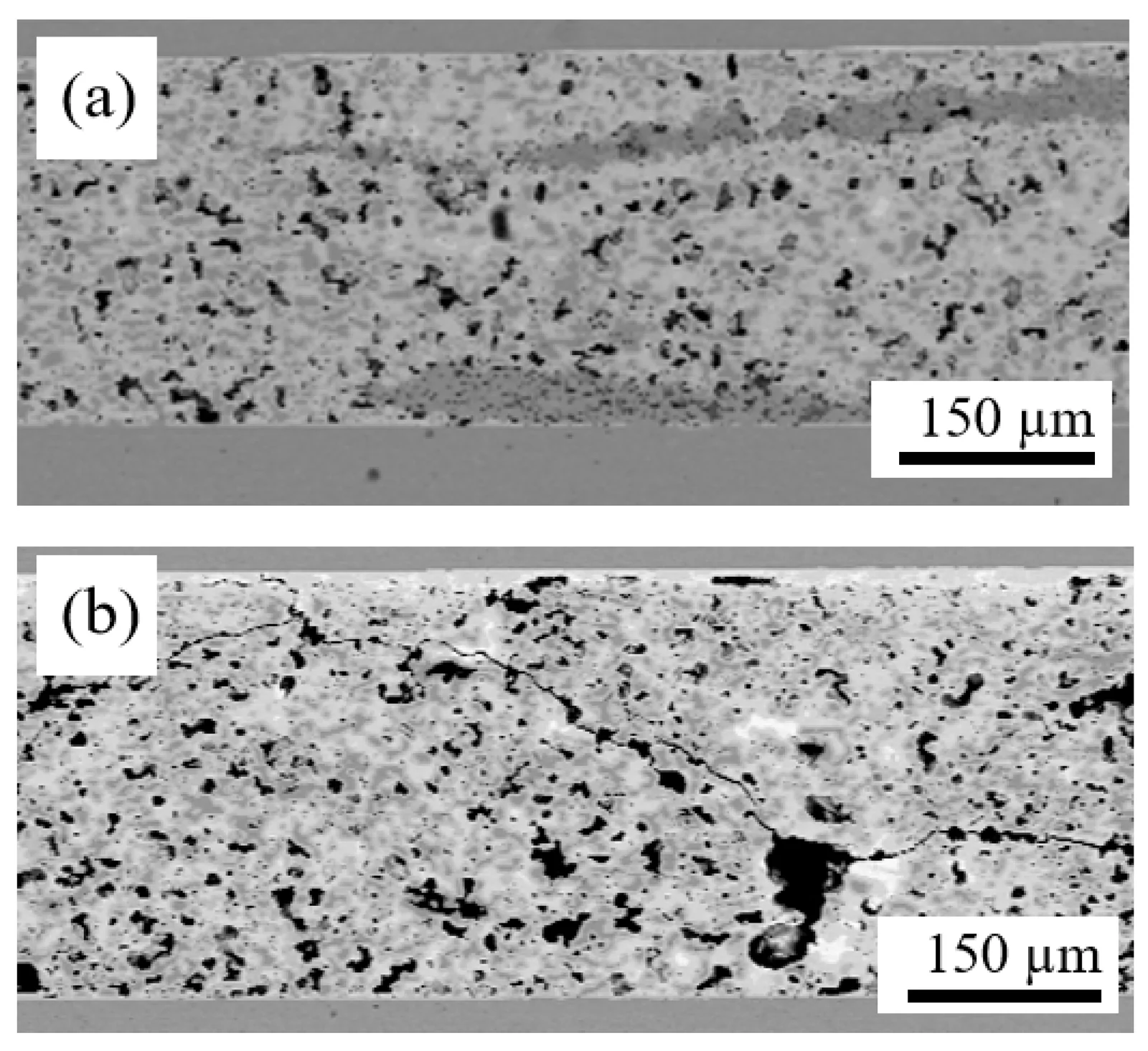
Microstructure and performance evolution of porous Cu-based interconnect bondlines before and after thermal shock: (**a**) microstructure before thermal shock; (**b**) microstructure after thermal shock.

The thermal shock test was designed to simulate an environment where devices are subjected to frequent start–stop cycles and abrupt temperature changes. By applying thermal cycle stress in a rapid alternating manner, this test focuses on investigating the fatigue failure resistance of the interconnection interface of porous Cu composite bondlines under severe temperature gradients and analyzes the laws of interfacial crack initiation and propagation, as well as the attenuation characteristics of interfacial bonding strength. As shown in Figure 9a,b, porous Cu/SAC305 interconnect bondlines presented outstanding anti-fatigue characteristics. After 1000 thermal shock cycles, only a small number of voids and cracks were generated in the microstructure, showing much better operational performance than pure SAC305 bondlines [42,43,44].

**Figure 10:**
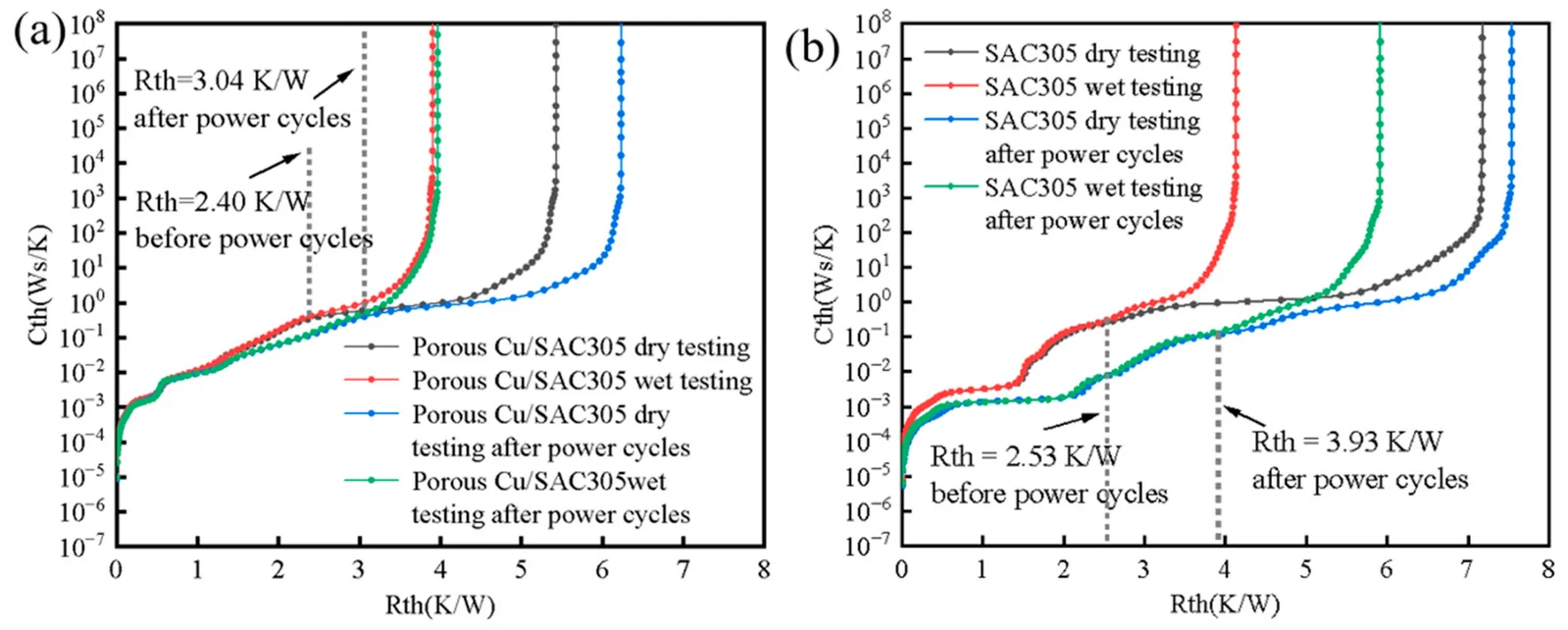
Thermal resistance evolution of different types of devices before and after power cycles [45]: (**a**) porous Cu/SAC305-packaged devices; (**b**) SAC305-packaged devices.

The power cycling test was designed to simulate an environment where devices undergo repeated power on–off cycles and periodic current loading. By applying periodic electrical power loads to induce internal temperature cycling and alternating thermal stress in these devices, this test focuses on investigating the damage evolution law of the interconnection interface of porous Cu composite bondlines under the coupled action of Joule heating and thermomechanical fatigue. Liu et al. packaged SiC diodes with porous Cu/SAC305 composite bondlines and subjected the packaged SiC devices to 30,000 cycles of second-scale power cycling tests at a constant 80 °C junction temperature. As presented in Figure 10a,b, the thermal resistance of the porous Cu/SAC305-packaged SiC devices increased from 2.40 K/W to 3.04 K/W after 30,000 cycles, while that of the SAC305-packaged counterparts increased from 2.53 K/W to 3.93 K/W [45]. In other words, compared with pure SAC305 solder, devices encapsulated with porous Cu/SAC305 composite solder exhibit lower thermal resistance and a slower degradation rate of thermal resistance during power cycling, which contributes to enhancing the long-term service thermal reliability of SiC devices.

## 5. Conclusions and Challenges

### 5.1. Conclusions

Porous Cu-based bondlines exhibit comprehensive advantages, including low costs, high thermal conductivity, excellent wettability, and designable pore structures, which well match the core requirements of low thermal resistance and high reliability for packaging interconnection materials in high-power electronic components. Accordingly, they have become a research hotspot in the field of electronic packaging interconnection. This work systematically summarizes the research progress on the preparation methods, interconnection methods, and reliability of porous Cu-based interconnection materials, with the main conclusions as follows:

(1) Four conventional fabrication methods for porous Cu, namely electrodeposition, dealloying, powder sintering, and oxidation–reduction, have respective merits and drawbacks. A novel preparation method using CuO as the matrix and NaCl as the pore-forming agent successfully realized the controllable fabrication of multi-gradient pore sizes.

(2) All three existing interconnection methods for porous Cu in electronic packaging demonstrate outstanding application performance. Porous Cu-reinforced solder bonding forms mechanical interlocking via the infiltration of molten solder into the porous Cu framework, which significantly suppresses the growth of Cu-Sn intermetallic compounds and Bi segregation, yielding a shear strength of 66.9–85.2 MPa. Transient liquid-phase bonding utilizes metallurgical reactions between low-melting-point solder and porous Cu to form high-melting-point intermetallic compounds, and the bondline strength remains at 73 MPa under shearing at 300 °C. Cu-Cu bonding eliminates the need for additional solder and forms a Cu-Cu interconnection structure through atomic thermal diffusion, making it suitable for high-temperature operating scenarios.

(3) Reliability tests, including high-temperature aging, thermal shock, and power cycling, have verified the excellent service stability of porous Cu interconnection bondlines. The bondlines maintain high interconnection strength after high-temperature aging, effectively preventing device failure. After 1000 cycles of thermal shock with a temperature difference ≥ 165 °C, only a small number of voids and cracks form, indicating far superior fatigue resistance compared to SAC305 bondlines. When applied to SiC diode packaging, the thermal resistance degradation rate of porous Cu/SAC305 composite bondlines is significantly lower than that of pure SAC305 bondlines after 30,000 rapid power cycles, greatly improving the thermal reliability of devices.

### 5.2. Challenges

Although remarkable progress has been made in the research on porous Cu-based interconnection materials, numerous critical technical challenges remain to be overcome for their translation from laboratory research to engineering applications. The core challenges are mainly reflected in the following three aspects:

(1) Precise fabrication of porous Cu with tailored pore sizes and porosities

Although the novel preparation method has realized controllable pore sizes in the range of 5–100 μm, the quantitative relationship between processing parameters (sintering temperature, pore-forming agent content, holding time, etc.) and pore characteristics (pore size, porosity, ligament thickness, and pore connectivity) across the full scale from nanometers to micrometers and macro-dimensions has not been fully established. Consequently, it is difficult to achieve precise and repeatable fabrication of porous Cu with specific pore structures for different packaging scenarios, and low production efficiency further severely restricts its large-scale engineering application.

(2) Bottleneck in the development of pressureless bonding technology for porous Cu

Current mainstream interconnection methods for porous Cu, such as transient liquid-phase bonding and Cu-Cu bonding, mostly rely on high-pressure hot-pressing sintering. This process imposes stringent requirements on packaging equipment and process control and tends to cause thermal damage and mechanical stress to fragile electronic components and packaging substrates, making it incompatible with high-precision and fragile device packaging. Pressureless bonding is a key technology for the practical application of porous Cu interconnection, yet related research is still in the initial stage. Achieving reliable interconnection of porous Cu under pressureless conditions via structural design, atmosphere regulation, and flux development, while ensuring interfacial bonding strength and thermoelectric performance, represents an urgent technical bottleneck.

(3) Lack of validation for long-term reliability of porous Cu bondlines

Current studies on porous Cu mainly focus on fabrication processes and short-term laboratory reliability assessments. However, the actual service environment of electronic devices is more complex and harsh, involving coupled effects of long-term high temperature, humidity, vibration, and electrochemical corrosion. Systematic long-term reliability tests of porous Cu bondlines under simulated service conditions and device-level application verification are still lacking. The microstructural evolution and degradation laws of mechanical and thermoelectric properties of porous Cu bondlines under long-term multi-factor coupling remain unclear. Furthermore, insufficient research has been conducted on the failure mechanisms of porous Cu bondlines, and no effective lifetime prediction model has been established to provide theoretical support for the engineering reliability design and evaluation of porous Cu-based interconnection materials.

## Figures and Tables

**Table 1:** Pore size/ligament distribution and advantages/disadvantages of porous Cu matrix prepared by different methods.

Preparation Method	Raw Materials	Diameter of Pore (μm)	Thickness of Ligament (µm)	Advantage	Weakness	Ref.
Electrodeposition	Polyurethane foam, CuSO_4_, H_2_SO_4_, NH_3_·H_2_O	140–680	30–170	Low cost, mechanical properties	Uncontrollable shape	[16]
		50–200	48.67–187.90			[17]
	CuSO_4_, HCl, CH_3_COOH	~17.68	~17.70			[18]
Dealloy	Cu-Zn, HCl	0.015–0.050	0.015–0.078	Adjustable porosity, nanopores	High cost, long preparation process, high brittleness	[19]
		0.019–0.021	0.018–0.030			[20]
	Cu-Zn, HCl, NH_4_Cl	0.037–0.130	0.030–0.176			[21]
	Cu-Al, HCl	~0.2	~0.2			[22]
	Cu-Al, H_3_PO_4_	~0.02–0.03	~0.02–0.03			[23]
	Cu-Al, NaOH	~0.015–0.044	~0.023–0.035			[24]
	Cu-Mn-Si, HCl	0.026	0.032			[25]
Powder Sintering	Cu particles	~9.506	~18.876	Low cost	Uncontrollable shape, High-temperature preparation	[26]
		~5.238	~12.098			[27]
Redox	Cu foil, S, H_2_	~3	~3	Adjustable porosity and pores, low cost	Long preparation process; high energy consumption	[28]
	CuO particles, H_2_	~5–100	~20–40			[29]

**Table 2:** Mechanical properties, advantages and disadvantages of porous Cu bondlines with different interconnection technologies.

Methods	Composition of Bondline	Remelting Point (°C)	Shear Strength (MPa)	Advantage	Weakness	Ref.
Porous Cu-reinforced solder bonding	Cu, SnBi, Cu_3_Sn, Cu_6_Sn_5_	138	66.9	Mechanical interlocking, excellent mechanical properties, inhibited IMC growth	Poor high-temperature resistance, internal voids	[30]
	Cu, SnBi, Cu_3_Sn, Cu_6_Sn_5_	138	85.3			[31]
	P-Cu, SAC305, Cu_3_Sn, Cu_6_Sn_5_	217	70.0			[32]
TLP bonding	Cu, Cu_3_Sn, Cu_6_Sn_5_	415	70.7	Low-temperature reflow–high-temperature service; excellent mechanical impact resistance	Complicated preparation process, interfacial voids	[29]
	Cu_3_Sn	676	/			[33]
	Cu, Cu_3_Sn, Cu_6_Sn_5_	415	83.0			[34]
Cu-Cu Bonding	Cu	1084	30.8	Excellent thermal and mechanical properties	Poor corrosion resistance, high processing difficulty	[35]
	Cu, Cu(Au)	1084	~40.0			[36]
	Cu	1084	22.1			[37]
	Cu	1084	~22.0			[38]
	Cu	1084	/			[39]

## Data Availability

No new data were created or analyzed in this study. Data sharing is not applicable to this article.
